# Evaluation of droplet digital PCR for the clinical diagnosis of toxoplasmosis

**DOI:** 10.1128/spectrum.02984-25

**Published:** 2025-10-28

**Authors:** Elise Recalt, Alexandra Lespagnol, Lya Hamet, Hélène Guegan, Jean-Pierre Gangneux, Marie-Dominique Galibert, Florence Robert-Gangneux

**Affiliations:** 1 Laboratoire de parasitologie et mycologie, Centre hospitalier Universitaire de Rennes 36684, Rennes, France; 2 Laboratoire de génétique somatique, Centre hospitalier Universitaire de Rennes 36684, Rennes, France; 3 Univ Rennes, CHU Rennes, Inserm, EHESP, Irset (Institut de Recherche en Santé Environnement Travail)-UMR_S 1085 27079, Rennes, France; Brown University, Providence, Rhode Island, USA

**Keywords:** toxoplasmosis, diagnosis, droplet digital PCR, *Toxoplasma gondii*

## Abstract

The diagnosis of active toxoplasmosis in fetus, newborns, and immunocompromised patients relies on molecular detection of the parasite DNA in various samples according to the clinical setting. The quality of molecular diagnosis is essential to guide specific treatment. Today, real-time PCR (qPCR) based on the REP-529 target is the reference method, but given the emergence of droplet digital PCR (ddPCR) in other diagnostic fields, it is essential to evaluate its clinical performance for the diagnosis of toxoplasmosis. In this study, we evaluated the Naica Crystal ddPCR system (Stilla Technologies) on 64 samples from patients with confirmed toxoplasmosis (30 congenital infection, 23 infections in immunocompromised patients, and 5 ocular toxoplasmosis) and 21 negative samples from patients without toxoplasmosis. Our results showed a concordance of 97% between qPCR and ddPCR, and a good correlation (*R*^2^ = 0.80) between the C*_T_* of the qPCR and the concentration in copies/µL obtained in ddPCR. However, we observed a matrix effect leading to a fluctuation of the positive threshold and impacting the separability score. Optimization of the ddPCR program according to the sample type is essential to ensure reliable interpretation.

**IMPORTANCE:**

To date, droplet digital PCR (ddPCR) contributes to the detection of infectious agents in the environment, but few studies have evaluated its interest in human diagnosis, particularly the Naica Crystal ddPCR. This study shows that it has similar performance to real-time PCR for the diagnosis of toxoplasmosis and could be of interest for parasite quantification, but its cost limits its use.

## INTRODUCTION

Toxoplasmosis is a common parasitic infection caused by the protozoan *Toxoplasma gondii*, which can persist lifelong in its host as cysts, preferentially located in the brain, the muscles, and the retina (1). Although asymptomatic in 90% of cases in immunocompetent patients, it can be life-threatening in immunocomromised patients. Two main patient populations are at risk of severe clinical forms: patients with a deficiency in cellular immunity and fetuses from mothers with primary-acquired toxoplasmosis during gestation (2). Today, accessible diagnostic tools are available to ensure the screening, diagnosis, and monitoring of these patients. Determining the serological status is the first step in assessing the risk of primary infection in pregnant women or solid transplant patients, or the risk of cyst reactivation in immunocompromised patients (3, 4). Direct search for parasite DNA by molecular methods is mainly indicated for antenatal or postnatal diagnosis of congenital toxoplasmosis, or to document *Toxoplasma* infection in immunocompromised patients presenting with ocular, neurological, or other clinical signs suggesting disseminated infection. It is also widely used to screen for *T. gondii* infection in patients with hematopoietic stem cell transplantation or solid organ transplantation to detect cyst reactivation early (5). The use of quantitative PCR (qPCR) targeting the repeated sequence REP-529 has greatly contributed to improving the sensitivity of diagnosis and thus patient care for toxoplasmosis (6). In recent years, droplet digital PCR (ddPCR) has shown its potential in oncology for the detection of mutations (7), but its interest in the diagnosis of infectious diseases remains to be demonstrated. A review published in early 2019 reported the application of ddPCR for the detection of protozoa, helminths, and arthropods (8), with potentially interesting prospects for malaria and schistosomiasis. The principle of ddPCR was described in the 1990s (9), but it has been more widely developed since 2010 (10). It often uses the same primers and probes as qPCR, but it differs from the latter by its amplification process, which is based on a fractionation of the amplification mix containing the pathogen DNA, into thousands of lipid droplets, each representing a distinct reaction chamber in which the sequence of interest is randomly incorporated and is amplified separately by PCR reaction during the thermocycling step. A rapid microfluidic analysis of the thousands of droplets makes it possible to separate the positive droplets from the negative ones thanks to their fluorescence and to convert the results into digital data. The absolute concentration of target DNA can be calculated using Poisson distribution statistics without the need for a standard curve (11). Various commercial systems are now available using chips or plates (12).

This technology has demonstrated its potential; however, its application for the detection of *T. gondii* remains very limited. One study highlighted its relevance for parasite detection and quantification (13), and another one explored its relevance in a veterinary context (14). Actually, ddPCR is a next-generation end-point PCR technique with high performance. It could be very useful for the rapid and sensitive detection of low concentrations of *T. gondii* DNA, enhancing sensitivity and precision in the detection and quantification of small amounts of pathogen DNA, while preserving all the advantages of qPCR.

The diagnosis of toxoplasmosis is a major public health issue around the world; thus the diagnostic potential of new technologies such as ddPCR must be assessed to determine their role within the patient care algorithm.

The aim of this work was to retrospectively evaluate the Naica Crystal ddPCR system (Stilla Technologies, Villejuif, France) for the diagnosis of active toxoplasmosis using banked DNA samples from patients with a known diagnosis.

## MATERIALS AND METHODS

### Patients and samples

**Fig 1:**
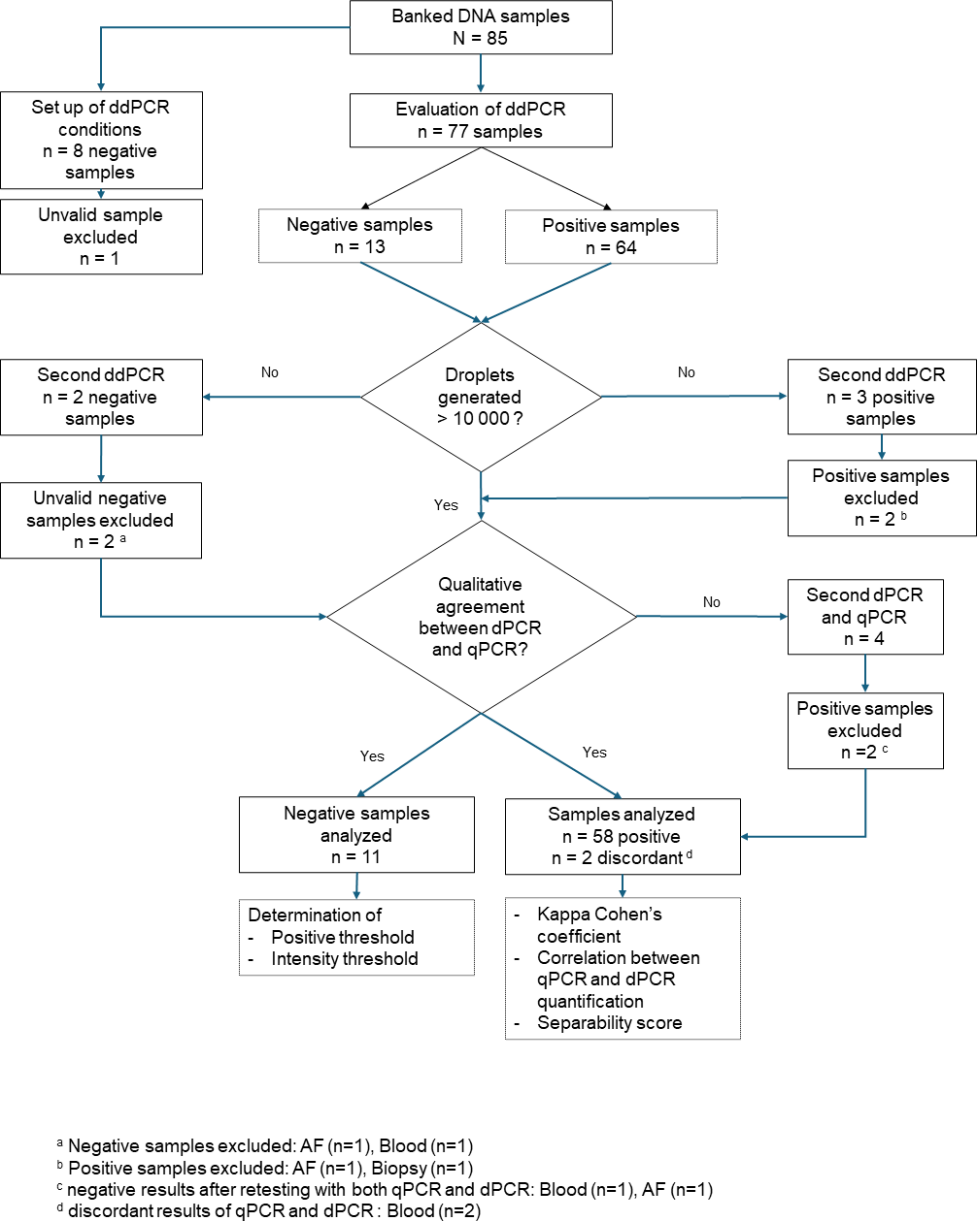
Study design and analysis of results.

We selected samples from the laboratory DNA bank, collected between 2011 and 2023 as part of investigations for acute or congenital toxoplasmosis using qPCR. The extracted DNA was stored at −20°C, and various DNA samples were selected according to the C*_T_* of initial qPCR and the specimen type. Overall, 85 samples were included, consisting of 21 negative DNA from patients with other diagnoses or for whom toxoplasmosis was ruled out, and 64 positive specimens from 58 patients with a diagnosis of toxoplasmosis documented by imaging and follow-up after specific treatment, or prenatal and postnatal follow-up in the framework of congenital toxoplasmosis screening (Fig. 1). The sample types positive for *Toxoplasma* qPCR were as follows: abscess and biopsy (*n* = 8), ocular samples (*n* = 6), bronchoalveolar lavage fluid (BALF) (*n* = 3), cerebrospinal fluid (CSF) (*n* = 3), amniotic fluid (AF) (*n* = 8), placenta (*n* = 22), and blood sample (*n* = 14). They were collected from 30 cases of congenital infection, 23 immunocompromised patients with cerebral or disseminated toxoplasmosis, and 5 immunocompetent patients with ocular toxoplasmosis. Negative samples from miscellaneous sample types were also included: AF (*n* = 9), pulmonary samples (*n* = 4), CSF (*n* = 3), blood (*n* = 2), urine (*n* = 2), and cornea scraping (*n* = 1).

### Real-time PCR

The initial diagnosis of the samples was performed using qPCR targeting REP-529 (GenBank AF146527) as previously described (15). The qPCR was not repeated for this study, except in the case of a discrepant result with ddPCR.

DNA extraction was carried out manually using the QIAamp DNA Mini Kit (Qiagen, Hilden, Germany) (from 2011 to 2019) or on EZ1 device using the DSP Virus DNA Extraction Kit (since 2020) (Qiagen). Quantitative PCR was performed using 5 µL of extracted DNA, TaqMan Universal PCR Master Mix containing uracil-DNA glycosylase (Applied Biosystems, ThermoFisher, Villebon-Sur-Yvette, France), and previously described primers and probes (15). Specific primers and probes for the IEC were also amplified in duplex. Amplification for 45 cycles and data analysis were performed using the QuantStudio 5 system (ThermoFisher) and the QuantStudio Design & Analysis Software 1.3.1, respectively. Positive results were expressed in amplification cycles (C*_T_*). All positive results up to 40 C*_T_* were considered valid, after checking the amplification curves.

Quantification in *Toxoplasma*/mL was performed using a standard curve obtained through 10-fold serial dilutions of a calibrated *Toxoplasma* suspension provided by the Molecular Diagnosis Section of the National Reference Center (NRC) for Toxoplasmosis (CHU Montpellier, France), although the quantification result is not routinely reported for diagnostic purposes. The lyophilized vial, prepared with *Toxoplasma* RH strain tachyzoites, was rehydrated with molecular grade water, following the NRC instructions, to obtain a suspension of 10,000 tachyzoites/mL (16).

### Digital PCR

The ddPCR system used was the Naica Crystal ddPCR system (Stilla Technologies) with a Sapphire chip, consisting of four identical microfluidic networks, allowing the analysis of four samples. Three chips can be run together, that is, 12 samples.

For this evaluation, the primers and probes used for routine qPCR (targeting *Toxoplasma* and the internal control) were used, in combination with the Naica 10× multiplex PCR Mix containing Buffer A and Buffer B (reference R10105). The reaction mix contained 12.5 µL of Naica multiplex PCR Mix, primers, and probe at a final concentration of 500 and 200 nM, respectively, 2.5 µL of IEC Mix, and 5 µL of DNA sample, in a final volume of 25 µL. After manual deposition of the reaction mix onto a Sapphire chip (Stilla Technologies), partitioning and thermocycling steps were performed using the Naica Geode system (Stilla Technologies). The ddPCR was conducted with the following program: partitioning at 40°C, enzymatic reaction for 2 min at 50°C, initial denaturation for 10 min at 95°C, followed by 45 cycles of 15 s at 90°C and 1 min at 60°C for hybridization and elongation. The ddPCR ended with 1 min at 40°C and a release step using the Sapphire V1 program. Fluorescence analysis of the chips and image acquisition were performed with the Naica Prism within 24 h after thermocycling. Data analysis and interpretation were carried out using the Crystal Miner software (Stilla Technologies) as previously described, and following the dMIQE recommendations (17, 18).

The ddPCR program was validated by passing two DNA samples of known concentration range (10^3^ and 10^4^ parasites/mL obtained from the dilution of the calibrated suspension) and eight negative AF samples (Fig. 1). Only chambers yielding more than 10,000 droplets were analyzed. The positivity threshold was determined by calculating the mean fluorescence of negative samples + 3 SD (automatically set by the Crystal Miner software or manually adapted on the 1D graphs). The remaining samples were run using these parameters. The separability score was provided directly by the analysis software, with a score >5 attesting a correct separation of positive and negative populations. Discordant results were rerun with qPCR and with ddPCR.

### Statistics

The concordance of ddPCR and qPCR results was assessed using Cohen’s kappa index, the value of which determines the strength of the agreement according to the following interpretation: <0.01 poor, 0.01–0.20 slight, 0.21–0.40 fair, 0.41–0.60 moderate, 0.61–0.80 substantial, and 0.81–1.0 almost perfect. A Spearman correlation test was performed to analyze the correlation between qPCR C*_T_* and ddPCR quantification results. A nonparametric Kruskal-Wallis test was used to compare the separability scores between samples. All statistics were performed using GraphPad Prism software version 9 (GraphPad Software, San Diego, CA, USA).

## RESULTS

### Validation of ddPCR conditions and result interpretation

**Fig 2:**
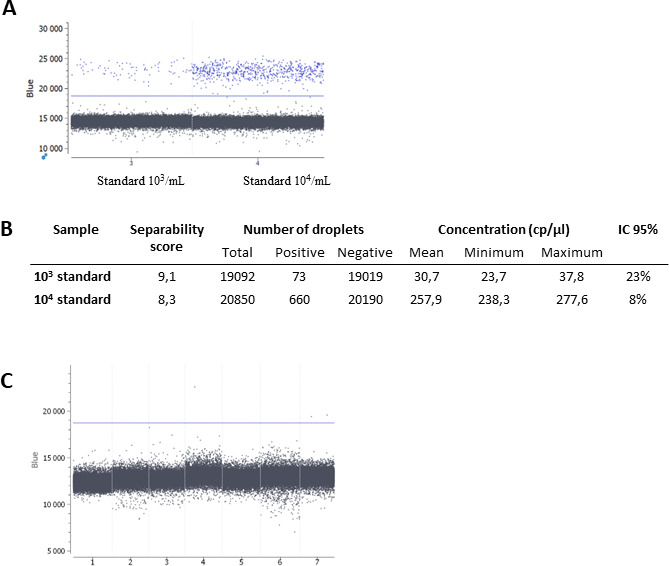
Validation of the ddPCR conditions. Separation of positive and negative droplets for REP-529 (**A**) on two *Toxoplasma*-calibrated standards. Quantification results acquired with ddPCR targeting REP-529 for both calibrated standards (**B**). Cumulative results of eight negative samples allowing to set the fluorescence threshold at 18,730 fluorescence units. Note that one positive droplet was observed above this threshold for several samples (**C**).

The ddPCR program was first validated with a chip containing the two calibrated DNA with known *Toxoplasma* concentration (10^3^ and 10^4^ parasites/mL) and two negative samples (water). The two targets REP-529 and IEC were detected on the dark blue and cyan channels, respectively, as shown for the two calibrated suspensions (Fig. 2A and B). The partition was successful with a total number of droplets produced >19,000 for both positive samples. The threshold was set automatically, and the separability score was >5 (Fig. 2C). The ratio between the two tested quantifications in copies/µL (30.7 and 257.9 copies/µL, respectively) was 8.4, which is close to the expected 10-fold ratio between the two tested concentrations (Fig. 2C). To validate the general conditions of interpretation, eight negative AF samples were further tested. One sample was re-assessed because the partition failed (<10,000 droplets) and was finally excluded, as it yielded similar results. Overall, the fluorescence intensity threshold was set at 18,730 fluorescence units for REP-529 and the positive threshold was defined as >2 positive droplets, as two out of seven analyzable negative samples presented one and two droplets above the cloud of the negative droplet population, respectively (Fig. 2D).

Following validation of these conditions, 64 positive samples and 13 remaining negative samples were run. The sample management is described in Fig. 1. Three positive samples (one AF, one blood, and one biopsy specimen) and two negative samples (one AF and one blood sample) did not meet the quality criteria (<10,000 droplets generated) and were re-assessed. Four samples failed again and were excluded from the analysis (Fig. 1). Overall, the partitioning step was successful in 80 out of 85 (94%) cases, taking into account both the validation step and the further blind evaluation study.

### Concordance of ddPCR and qPCR

At first testing, four positive samples were found to be discordant between the ddPCR and initial qPCR results, thus a new qPCR was performed to check results. Two DNA samples were negative when the qPCR was repeated, thus they were excluded from analysis, while the remaining two samples were confirmed positive (C*_T_* = 37 and 39, respectively). A new ddPCR was carried out with the two remaining false negative samples, after dilution to 1:10 and 1:50, but the result was still negative. No false positive results were obtained with ddPCR. Finally, the interpretation of results was carried out on 71 samples including 11 negative samples and 60 positive samples (Fig. 1). The concordance of ddPCR and qPCR was 97.2% (69/71) with a Cohen’s kappa index of 0.93 confirming an “almost perfect” agreement of the results between the two techniques (Table 1). Detailed results by sample type are shown in Table 2.

**TABLE 1:** Concordance table of ddPCR and qPCR results (*n* = 18 negative and *n* = 60 positive results)

qPCR result	ddPCR		Total
	Negative	Positive	
Negative	11	0	11
Positive	2	58	60
Total	13	58	71

**TABLE 2:** ddPCR results obtained for 78 interpretable samples, according to sample type

Samples	qPCR: C*_T_* (mean ± SE)	ddPCR			
		No. of droplets (mean ± SE)	No. of positive droplets (mean ± SE)	Concentration (copies/µL) (mean ± SE)	Separability score (mean ± SE)
Positive samples					
Biopsy (*n* = 7)	22.7 ± 3.6	20,641 ± 1,713	8,111 ± 9,503	9,025 ± 13,981	7.6 ± 1.1
Aqueous humor (*n* = 6)	33.5 ± 5.5	21,004 ± 1,037	1,377 ± 3,294	644 ± 1,548	7.3 ± 2.4
BALF (*n* = 3)	31.3 ± 7.4	21,733 ± 2,650	496 ± 826	215 ± 360	4.3 ± 1.5
CSF (*n* = 3)	31 ± 1,0	18,853 ± 1,636	166 ± 214	76 ± 102	8.1 ± 2
Amniotic fluid (*n* = 6)	31.8 ± 2.2	20,725 ± 1,524	303 ± 659	119 ± 257	6.2 ± 2.3
Placenta (*n* = 22)	32.3 ± 4.3	21,285 ± 2,177	682 ± 2,962	461 ± 2,077	4.7 ± 1.9
Blood/bone marrow (*n* = 11)	32.2 ± 5.1	21,034 ± 1,208	131 ± 415	60 ± 182	4.5 ± 2.3
Negative samples					
Liquids^*a*^ (*n* = 9)	NA^*d*^	20,984 ± 1,156	0.4 ± 0.7	NA	NA
Pulmonary samples (*n* = 4)	NA	20,807 ± 587	0.25 ± 0.5	NA	NA
CSF (*n* = 3)	NA	21,273 ± 1,629	0	NA	NA
Other^*b*^ (*n* = 2)	NA	20,050 ± 1,090	0	NA	NA
Discordant results					
Blood	40/37^*c*^	18,720	0	NA	NA
Blood	33/39^*c*^	20,123	0	NA	NA

^*a*^ Amniotic fluids (*n* = 7) and urine (*n* = 2).

^*b*^ Blood (*n* = 1) and cornea scraping (*n* = 1).

^*c*^ First and second qPCR results after retesting.

^*d*^ NA, not adapted.

### Correlation of quantitative results of ddPCR and qPCR

**Fig 3:**
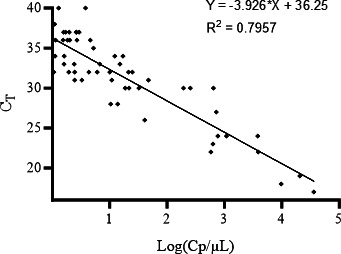
Correlation between qPCR and ddPCR results, expressed in C*_T_* and copies (Cp)/µL, respectively.

There was a good correlation between C*_T_* results of qPCR and the concentration values obtained with ddPCR in copies/µL (*R*^2^ = 0,80, *P* < 0.001), considering that most assays were carried out on low to very low parasite concentrations (Fig. 3).

Results obtained with the calibrated standards 1,000 and 10,000 *Toxoplasma*/mL displayed approximately 30,000 and 258,000 copies/mL (Fig. 2B), which is consistent with recent studies evaluating the copy number of *REP*-529 per genome. In a recent study, Nabet et al. (13) estimated the number of REP-529 copies from 14 to 160 in genotype II strains, by comparison to the α-tubulin single-copy gene.

### Separability score

**Fig 4:**
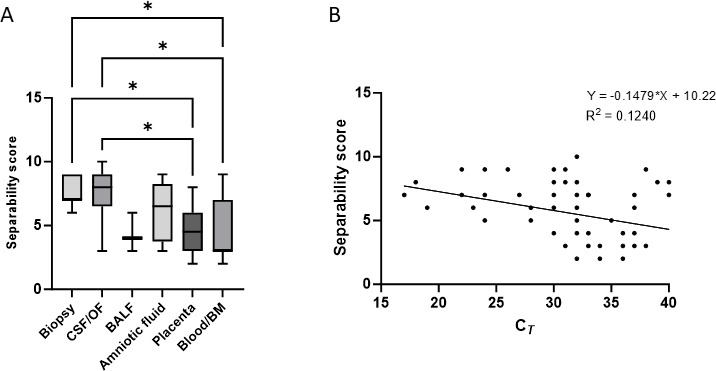
Mean separability score observed for various matrices (**A**). Correlation between separability score and qPCR C*_T_* result (**B**). *P < 0.05.

Low mean separability scores (<5) were observed for some matrices (BALF, placenta, and blood) (Fig. 4A). The difference in separability score was significant among the different sample types (*P* = 0.0008, ANOVA) and did not appear to be related to the parasite load of the sample, as shown on Fig. 4B, where a poor correlation can be observed (*R*^2^ = 0.124). Separability scores differed individually between biopsy samples and placenta or blood (*P* < 0.05) and between CSF/ocular samples and placenta or blood (*P* < 0.05) (Fig. 4A).

## DISCUSSION

To date, the use of ddPCR to detect pathogens has mainly been evaluated for veterinary purposes or environmental detection (19–21). Application in human diagnosis is still scarce, but nice studies have shown its interest for *Plasmodium* diagnosis or drug resistance (22, 23) as well as SARS-CoV2 diagnosis (24), and its potential in infectious diseases is probably underestimated. In this study, we evaluated the Naica system, rarely used in the field of infectious diseases, for the detection of *T. gondii*. Our study highlights a good concordance and a strong correlation between ddPCR copy numbers and qPCR C*_T_*, using samples from different matrices. These results are in agreement with literature data for other viruses or parasites (25–27).

However, many performance criteria must be taken into account to validate the ddPCR program (18), particularly when a wide range of sample types are used to diagnose infection, thus the technique needs to be thoroughly standardized to yield reliable results for human diagnosis and avoid false positive results, as one or two positive droplets can be observed in negative samples, as observed here (13). We experienced gaps with the partitioning step, which failed for five samples (6%). Such failures are described by the manufacturer and can be due to the presence of air bubbles, traces of detergent, and/or artifacts. These defects can result from the manual loading step and might be avoided by the training and experience of the operator, but we found that they occurred randomly throughout the series, and even after retesting by a different operator, suggesting a sample-specific problem. These failures increase the cost of the assay. Additionally, we also observed that two qPCR-positive samples, for which PCR inhibitors were suspected through IEC results, tested repeatedly negative with ddPCR despite dilution to 1:10 and 1:50. Although ddPCR is less sensitive to inhibitors, it would not be completely immune to it (28). These samples were blood samples from immunocompromised patients with disseminated toxoplasmosis, with low parasite loads. It is well-known that qPCR sensitivity can be challenged by the presence of inhibitors, which are more often associated with biopsy samples or placentas (29), and can be detected through the observation of inadequate gap in IEC C*_T_* results on plain and diluted sample DNA. We also highlighted a significant matrix effect on ddPCR, which resulted in variations in the fluorescence intensity threshold for negative populations. As a result, the application of an automated threshold allowing the distinction between positive and negative elements is not possible. This constraint imposes the application of a manual threshold and induces an operator bias. This drawback could be avoided by running a single type of matrix on a given chip, including a negative sample and a standardized positive sample, which could make it easier to determine a threshold, but would double the price of the test, already high (at least 10-fold higher than a qPCR reaction) not to mention the cost of control samples, and the number of runs.

For some samples (BAL, blood, and placenta), we encountered difficulties in distinguishing positive and negative droplet populations, linked to a “rain” effect. Despite an attempt to modify the probe concentrations, we did not observe any variation in droplet fluorescence, nor clearer separation. This effect results in low separability scores for some samples and seems to be linked to the sample matrix and not to the parasite load. Studies indicate that rain effect often results from poor ddPCR settings (30) or the presence of inhibitors (31). They suggest that a higher number of cycles, longer elongation times, associated with adequate temperatures could reduce this effect and improve the separability of the droplets (32). However, in our study, it was clearly associated with a few placentas or BAL samples, suggesting an effect linked to high-cellularity samples. A poor separability score did not appear to be linked to the duration of storage of DNA sample (data not shown), thus could unlikely be explained by sample degradation.

Quantification is not essential for the diagnosis of acute or congenital toxoplasmosis, but monitoring of the parasite load is a good indicator of treatment effectiveness (33) and could be contributive for early prognosis of congenital toxoplasmosis, although other parameters such as time of infection, parasite genotype, and delay to treatment play an important role in the establishment of fetal sequelae (34). The good correlation between the C*_T_* of qPCR and ddPCR absolute quantification is an encouraging indicator, which, coupled with the lower sensitivity of ddPCR to inhibitors, could help approach the quantification of parasite loads more reliably. A recent study underlined the advantages of ddPCR for HIV quantification, due to the needlessness of standard curve and lower variations than qPCR (35). However, the much lower *Toxoplasma* loads, compared to viral loads, make indispensable the use of repeated-sequence targets. The rep-529 sequence target has been widely adopted, as it is the most repeated described target to date. The first estimation of the copy number of REP-529 element by Homan et al. (36) was initially 200–300 per parasite genome, using southern blotting. However, in more recent studies, the number of REP-529 copies per *Toxoplasma* genome has been shown to be lower (13, 37). Indeed, Costa and Bretagne described a number of copies ranging from 21 to 68, by comparison to the single-copy gene *P30*, according to the parasite genotype (37).

Although absolute quantification was not the object of our study, we observed an expected 10-fold ratio between the two tested parasite concentrations. Indeed, the quantification results obtained for the calibrated standards, that is, 1,000 and 10,000 parasites/mL were 30,000 and 258,000 copies/mL, respectively, which was in full agreement with a copy number ranging from 21 to 30 per genome, as described by Costa and Bretagne for a type I strain (37). However, parasite quantification has not proved useful for patient management of toxoplasmosis until now, as the prognosis of congenital toxoplasmosis is mainly correlated to the trimester of gestation at fetal infection and to the parasite genotype. Nonetheless, ddPCR absolute quantification of *T. gondii* could be useful in other clinical scenarios, such as examining pathogen clearance in severely immunocompromised patients with poor response to treatment, and standardizing parasite load measurements across laboratories. In practice, ddPCR did not outperform qPCR, which remains the best screening tool with a high cost-benefit ratio.

### Conclusion

Overall, crystal ddPCR is an emerging technique in microbiology, whose performance could be interesting for the detection and quantification of *T. gondii*. However, the diversity of sample types used in clinical diagnosis requires complex adjustments for optimization of the Stilla Crystal system, and its cost remains a barrier for routine use, compared to the Bio-Rad system.

## Data Availability

The data can be accessed at the following link: https://doi.org/10.5281/zenodo.17422108.
